# Tuning mitochondrial dynamics for aging intervention

**DOI:** 10.1093/lifemedi/lnae008

**Published:** 2024-02-08

**Authors:** Xudong Yao, Xiaojun Xia, David C Hay, Mike Shipston, Hongwei Ouyang

**Affiliations:** International School of Medicine, International Institutes of Medicine, The Fourth Affiliated Hospital of Zhejiang University School of Medicine, Yiwu 322000, China; Dr. Li Dak Sum & Yip Yio Chin Center for Stem Cells and Regenerative Medicine, Department of Orthopedic Surgery of the Second Affiliated Hospital, Zhejiang University School of Medicine, Hangzhou 310058, China; International School of Medicine, International Institutes of Medicine, The Fourth Affiliated Hospital of Zhejiang University School of Medicine, Yiwu 322000, China; Centre for Regenerative Medicine, Institute for Regeneration and Repair, University of Edinburgh, Edinburgh EH1 1LT, United Kingdom; Biomedical Sciences, College of Medicine & Veterinary Medicine, University of Edinburgh, Edinburgh EH1 1LT, United Kingdom; Liangzhu Laboratory, Zhejiang University, Hangzhou 311121, China; Dr. Li Dak Sum & Yip Yio Chin Center for Stem Cells and Regenerative Medicine, Department of Orthopedic Surgery of the Second Affiliated Hospital, Zhejiang University School of Medicine, Hangzhou 310058, China; Key Laboratory of Tissue Engineering and Regenerative Medicine of Zhejiang Province, Zhejiang University School of Medicine, Hangzhou 310058, China; Department of Sports Medicine, Zhejiang University School of Medicine, Hangzhou 310058, China; China Orthopedic Regenerative Medicine Group (CORMed), Hangzhou 310058, China

**Figure 1.**
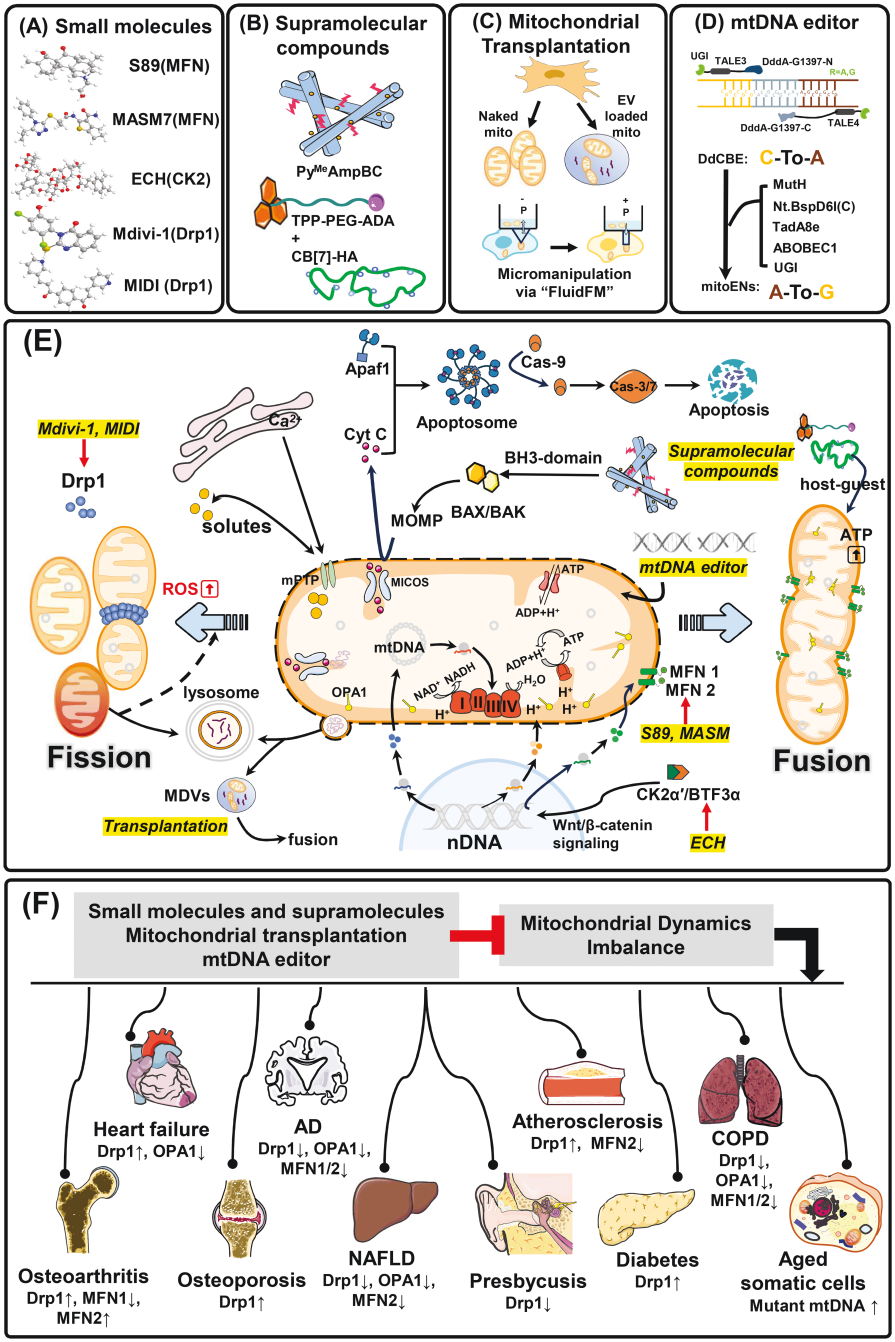
**Tuning mitochondrial dynamics to ameliorate mammalian aging.** (A) Small molecules: S89 and MASM7 activate mitochondrial fusion by targeting MFN1 and MFN2. Mdivi-1 and MIDI inhibit conformational changes of Drp1 to prevent fission. ECH activates the Wnt/β-catenin signaling pathway to promote MFN transcription and mitochondrial fusion. (B) Supramolecular compounds: Py^Me^AmpBC triggers MOMP to release cytochrome C from mitochondria into the cytosol. Subsequently, the assembled apoptosome recruits Cas-9 to induce the caspase cascade and apoptosis. TPP-PEG-ADA cooperates with CB[7]-HA, inducing fusion via host-guest chemistry. (C) Mitochondrial transplantation: both naked mitochondria and mitochondria loaded in extracellular vehicles are available. Exogenous mitochondria not only play functions but also fuse with recipient mitochondria which have been visualized by micromanipulation via “FluidFM” approach. +P, positive pressure; −P, negative pressure (D) mtDNA editor: a TALE combined with a DddA clip editor, DdCBE, allowing C-to-A editing. By introducing either MutH or Nt.BspD6I(C), along with TadA8e or ABOBEC1 and UGI, results in A-to-G editing. (E) Schematic depiction of utilizing approaches (A)–(D) to control mitochondrial fission and fusion: The outer mitochondrial membrane (OMM) interacts with the endoplasmic reticulum and lysosomes. Mitochondria release mitochondrial-derived vesicles to interchange mitochondrial cargo complexes. Membrane-shaping proteins including the mitochondrial contact site and cristae organizing system (MICOS) shape the IMM into cristae, which communicates with OMM and IMM through the outer membrane proteins and optic atrophy protein 1 (OPA1). The mitochondrial matrix is surrounded by OMM and IMM, which is the site of the ATP synthase, Ca^2+^ storage, mtDNA replication, and protein biosynthesis. While fission/fusion dynamic balance is fragile, aging-accompanied ROS accumulation stimulates excessive fission, disables OCR function, and causes an imbalance in Ca^2+^ level. (F) Mitochondrial dynamics imbalance contributes to the onset of several age-related diseases, presenting abnormal fission/fusion protein concentrations and mutated mtDNA as followed: Heart failure, Drp1↑, OPA1↓; AD, Drp1↓, OPA1↓, MFN1/2↓; Atherosclerosis, Drp1↑, MFN2↓; COPD, Drp1↓, OPA1↓, MFN1/2↓; Osteoarthritis, Drp1↑, MFN1↓, MFN2↑; Osteoporosis, Drp1↑; NAFLD, Drp1↓, OPA1↓, MFN2↓; Presbycusis, Drp1↓; Diabetes, Drp1↑; Aged somatic cells, mutant mtDNA↑. Pharmacological modulation of Drp 1 or MFN by small molecules and supramolecules, delivery of intact isolated mitochondria by transplantation, or the use of mtDNA editor to correct pathogenic mtDNA mutations, can be effective strategies to prevent aging-related cellular and organismal decline. ↑: up-regulation, ↓: down-regulation. AD, Alzheimer’s disease; COPD, chronic obstructive pulmonary disease; NAFLD, nonalcoholic fatty liver disease.

The mitochondrion is a double membrane structure within the cytoplasm that contains its own genome and generates the majority of the cell’s energy via aerobic respiration. Mitochondria naturally eliminate pathogenic mitochondrial DNA (mtDNA) mutations and repair dynamic architectures by controlling organelle division and fusion via guanosine triphosphatase (GTPase) dependent signaling. In this process, fusion compensates partially damaged mitochondria, whereas fission generates new mitochondria and dilutes the fraction that is dysfunctional. It is known that defects in GTPase-driven biogenesis cause dysfunctional oxidative phosphorylation and this is associated with mammalian aging and organ failure. Therefore, effectively targeting mitochondrial quality has the potential to rejuvenate cellular biology and ameliorate aging-associated disease (Fig. 1).

The GTPases Mitofusins 1 and 2 (MFN1 and MFN2) represent important targets in mitochondrial disease as they initiate mitochondrial membrane fusion (Fig. 1E). Indeed, a hallmark of myocardial aging is the accumulation of dysfunctional mitochondria due to non-redundant functions of MFN1 and 2. To target MFN1 fusion activity, a small molecule agonist was recently developed. Termed S89, it rescued mitochondrial fragmentation and swelling following ischemia/reperfusion injury by interacting with the GTPase domain of MFN1 [1], thus delayed aging-derived senescence resulting from mitochondrial DNA mutations, the oxidative stress induced by paraquat, iron-induced death and even neuromuscular disorder Charcot-Marie-Tooth disease type 2A caused by gene mutations. To modulate MFN2’s fusogenic activity, a further peptidomimetic small molecule, MASM7, was recently discovered (Fig. 1A). MASM7 activates MFN2 pro-tethering conformation and enables mitochondrial fusion resulting in increased membrane potential, mitochondrial respiration, and subsequent ATP production, providing promise to reduce age-related degenerative metabolic disease [2]. It should be noted that the direct targeting of MFN proteins is not the only way to boost mitochondrial fusion. Small molecules such as echinacoside (ECH), which drive casein kinase 2 *α*ʹ subunit heterodimerizing with basic transcription factor 3, can induce MFN2 expression. This has therapeutic potential and demonstrated beneficial effects in Alzheimer’s (AD) and Parkinson’s disease (PD) [3].

The regulation of mitochondrial fission in human aging has also been studied. The GTPase dynamin-related protein 1 (Drp1) uniquely triggers mitochondrial fission by chemoenzymatically constricting the mitochondrial surface to divide the organelle leading to mitophagy (Fig. 1E). Uncontrollable Drp1 activation leads to hyper-fragmentation, sustained opening of mitochondrial permeability transition pores and eventually apoptosis, which is commonly detected during aging. The most successful Drp1 inhibitor is Mdivi-1, a derivative of quinazolinone, which has been widely reported to mitigate disease, from myocardial failure to abnormal neurodegeneration (Fig. 1A). Additionally, Mdivi-1 assists T cell immunotherapy by restoring the expression of histocompatibility complex class I (MHC-I) in cancer, contributing to anti-tumor efficiency [4]. However, the lack of tissue specificity and off-target effects has limited the clinical use of Mdivi-1 inhibitors. Most recently, a new covalent molecule named MIDI was discovered [5]. As the structure of MIDI includes a double (pyridin-3-yl) propanone-substituted benzene and a double bond of the α,β-unsaturated ketone, MIDI interacts with Drp1 cysteines and effectively blocks Drp1 recruitment instead of directly targeting its tetramerization and GTPase activity. This provides a fresh angle to further establish Drp1 inhibitors that target age-related diseases.

In addition to those small molecules, a proof-of-concept supramolecular strategy has been attempted to balance mitochondrial homeostasis (Fig. 1B). A supramolecular antagonist was created by assembling a mitochondrial-responsive pentapeptide Py^Me^Amp with a peptide BH3 and an alkaloid camptothecin. BH3 depressed anti-apoptotic proteins Bcl-xL at the outer mitochondrial membrane (OMM) while camptothecin strengthened the release of mitochondrial cytochrome C, programming to cell death [6]. Inspired by this invention, *in vivo* tumor progression was tested. Supramolecular complex demonstrated robust bioavailability and altered mitochondrial fusion and fission dynamics in cancerous cells, potentially serving as mitochondrial targets in cancer caused by aging. Moreover, “artificially-induced mitochondrial fusion” under chemical stimulation, was first achieved using supramolecular polymerization [7]. This differs from fundamentally modulating mitochondrial GTPases. A polyethylene glycol backbone was linked with a triphenylphosphonium head and an adamantane tail (TPP-PEG-ADA) and grafted to hyaluronic acid (CB[7]-HA) to aggregate individual mitochondria through host-guest chemistry. Both *in vitro* and *in vivo*, this supramolecular system exhibited safety and efficacy in cell safeguarding and rehabilitating abnormal neurons.

Intact mitochondria natively exchange between cells in health and disease. Therefore, the transplantation of mitochondria may have a role to play in correcting age-related dysfunction. In support of this, the delivery of either naked exogenous mitochondria derived from mesenchymal stem cells, or muscle tissue derived isolated-mitochondria or mitochondria encapsulated in extracellular vesicles extracted from cardiomyocytes, have been shown to reverse low levels of metabolism in adipose tissue, brain, kidney, spleen, and heart [8]. However, the fate of donor mitochondria in subsequent cytoplasmic networks of recipient cells remains unknown. The latest innovative micromanipulation, known as “FluidFM” (Fig. 1C), has allowed the visualization of mitochondrial immediately participating in fusion within 20 minutes of transplantation and lasting for at least 14 days [9]. This is supported by numerous human clinical trials successfully using autologous or allogeneic mitochondrial transplantation which supports the notion of “aged-mitochondria replacement therapy”.

Another critical component of mammalian aging highlights a role for pathogenic mutant mtDNA. Using the human mitochondrial genome database MitoMap, over 90% of clinically identified mtDNA mutants highlighted are C/T and A/G transversions. However, it is difficult to imagine this as a clinically tractable approach as CRISPR-based genome editing rarely takes place in the inner mitochondrial membrane (IMM). Therefore, an alternative, protein-based editing platform was initially created in 2020, termed DddA-derived cytosine base editor (DdCBE) that enables mitochondrial C/T targeting (Fig. 1D). Recently, a transcription activator-like effector (TALE) integrating a nickase and a deaminase efficiently catalyzed A/G conversion in mtDNA, showing up to 77% frequencies without off-target effects [10]. Delivery of these mtDNA-based editors functionally corrected ATP content and oxygen consumption rates (OCRs) in multiple disease-relevant cell lines (Fig. 1E), however, *in vivo* evaluation and validation have yet to be tested.

In summary, the use of small molecules, supramolecular polymers, intercellular mitochondrial trafficking, and mtDNA genome editing are promising preclinical strategies to correct cellular vitality and metabolism during aging (Fig. 1F). However, the biggest obstacle in translating mitochondrial dynamics-targeted therapeutics between animal models and human clinical trials is the presence of long-journey to intracellular mitochondria, not only considering the desired cellular penetration at the expense of an increased risk of systemic toxicity but also providing the specific and precise mitochondrial targeting. A holistic perspective of how different dysfunctional mitochondria density interacts on diverse spatial and temporal scales to drive the cellular aging is necessary to understand and guide next treatment strategies. The field of mitochondrial correction is still in its infancy, we anticipate that this field offers great potential for the future treatment of chronic disease and human aging.
